# Physical Activity Interventions Framed by the Health Action Process Approach for Adults with Long-Term Conditions: A Scoping Review

**DOI:** 10.1007/s12529-024-10305-2

**Published:** 2024-07-15

**Authors:** Amy L. Silva-Smith, Coral L. Hanson, Lis Neubeck, Anne Rowat, Sheona McHale

**Affiliations:** 1 Helen and Arthur E. Johnson Beth-El College of Nursing and Health Sciences, University of Colorado at Colorado Spring, 1420 Austin Bluffs Parkway, Colorado Springs, CO USA; 2 Centre for Cardiovascular Health, Edinburgh Napier University, Sighthill Campus, Edinburgh, EH11 4DN UK; 3 School of Health and Social Care, Edinburgh Napier University, Sighthill Campus, Edinburgh, EH11 4DN UK; 4 Nursing & Health Care School, University of Glasgow, 57/504 Oakfield Avenue, Glasgow, G12 8LL UK

**Keywords:** Physical activity, Long-term conditions, Intervention, Health action process approach

## Abstract

**Background:**

Interventions that use the Health Action Process Approach (HAPA) model show promise for increasing PA frequency, duration, and intensity. However, there is limited understanding of how HAPA model variables have been operationalized for PA interventions in chronic disease to promote behavior change and sustained PA or whether the phase or continuous form of the HAPA model was used. The aim of this scoping review is to describe how the HAPA model variables for PA interventions were operationalized and provide details of implementation.

**Method:**

We searched five databases to identify studies published between January 1992 and March 2024. We aimed to describe (1) the characteristics of interventions including setting, delivery mode, duration, and content; (2) which HAPA variables were operationalized and the strategies used; and (3) the physical activity measures and outcome effects.

**Results:**

The search identified 23 interventions in 30 papers (12 protocols, 3 quasi-experimental studies, and 15 randomized controlled trials (RCTs)). Seven of the 15 RCTs reported significant positive effects of the HAPA model on PA behavior outcomes. Interventions operationalized between three and nine HAPA constructs showed significant variability in how the HAPA model is used in intervention research. PA measures varied from self-report to validated objective instruments.

**Conclusion:**

We found a lack of clarity in decisions about which HAPA constructs were included in interventions. The wide variability in operationalized HAPA constructs made it challenging to compare interventions. Researchers should provide more detail about intervention design and implementation procedures to enhance transparency.

**Supplementary Information:**

The online version contains supplementary material available at 10.1007/s12529-024-10305-2.

## Background

Globally, long-term conditions account for 41 million deaths each year [1]. Long-term conditions such as cardiovascular and respiratory diseases, diabetes, and cancer cause disabilities which impact individuals’ full engagement in activities of daily living, employment, and social activities [2]. Regular moderate-intensity physical activity (PA) can reduce morbidity and mortality in people who live with long-term conditions [3]. Physical activity is any body movement that involves skeletal muscle to produce energy expenditure beyond resting levels and is known to have beneficial effects on improving disease complications, physical function, and enhancing quality of life [4]. The initiation of PA, or ability to maintain PA, is affected negatively by long-term conditions resulting in higher mortality and morbidity and an increase in multi-morbidities [5]. To address these challenges, behavior change theories are used to design interventions to increase PA uptake in persons with chronic health conditions [6].

## Overview of the HAPA Model

There is a growing body of PA research reporting use of the Health Action Process Approach (HAPA) model, a social cognitive framework, as the model for behavior change interventions [7, 8]. The HAPA model addresses the intention-behavior gap through a two-phase process. The motivation phase comprised intention formation (goal setting) using risk perception, outcome expectancy, and perceived self-efficacy for the action (PA, for example). The motivation phase represents a mindset of building intention toward action (behavior change). Establishing the intention to act moves the individual’s mindset to the volition (goal pursuit) or action phase of the model. Bridging intention to action requires self-regulatory activities to initiate action, maintain (persist) action, and recover from interruptions in PA. In the volition phase, self-regulation includes action control (self-monitoring activities to increase focus), maintenance (coping) self-efficacy, and recovery self-efficacy. Initiation of the action requires action planning including determination of when, where, and how of the behavior and action control which includes self-monitoring designed to increase focus on the behavior to avoid distraction, temptation, and negative emotions. To persist and sustain the behavior, coping planning must also be undertaken to establish behavioral strategies to anticipate barriers and situations that may arise during the action phase [7, 9].

**Fig. 1:**
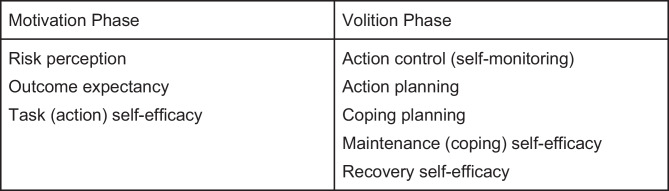
Constructs associated with each HAPA phase

The HAPA model incorporates 10 constructs (action control; action planning; coping planning; goal setting; intention; maintenance self-efficacy; outcome expectancy; risk perception; recovery self-efficacy; and task self-efficacy) and provides a basis for understanding how to target interventions most effectively based on stage-specific developmental needs (Fig. 1) [7–9]. The HAPA model has been applied as both continuum and stage processes. If the health behavior change is viewed as an ongoing process, then the continuum model is used. When used as a continuum model, HAPA is used to explain and predict behavior change. However, if the view is that individuals may have different mindsets depending on where they are in their course of health behavior change, the stage model applies. The three stages are pre-intenders (not yet through the motivational phase), intenders (ready to take action), and actors (engaged in the behavior). The stage is used to target intervention activities that move the individual further toward the desired behavior change. Combining the stage and continuum approaches is feasible and may provide more specificity for intervention development [7]. Whether used as a stage or continuum model, a growing body of literature provides evidence for interventions that use the HAPA model to design behavior change interventions [10–14].

In 2019, a systematic review with meta-analysis, including 95 papers, reported positive correlations with small to medium effect sizes among HAPA constructs and health behaviors (PA and/or nutrition). Each of the studies included at least four of the model variables [15]. Samples were identified as “clinical” (having a chronic condition) or “non-clinical” (e.g., student) but conditions for “clinical” samples were not clearly specified [15]. Consequently, applying these findings to PA intervention development and implementation for a specific patient population or long-term condition is difficult. To address this challenge and to provide the details needed by researchers and interventionists, we conducted a review of intervention characteristics for PA interventions using the HAPA model in long-term conditions.

## Methods

### Aims

The aims of this review were to describe (1) the elements/components of the interventions; (2) how HAPA variables were operationalized in interventions; and (3) the PA measures used and outcome effects for RCTs and quasi-experiments. Long-term conditions were selected as the focus to eliminate confounding contexts of recovery and rehabilitation inherent between acute and chronic conditions.

### Design

The scoping review followed the Joanna Briggs Institute (JBI) framework [16, 17]. Reporting was based on the Preferred Reporting Items for Systematic reviews and Meta-analyses extension for Scoping Reviews (PRISMA-ScR) [17]. By their definition, scoping reviews are systematic and utilize a mapping approach. The authors registered the review on Open Science Framework (October 28, 2021) [18]. This article does not contain any studies with human participants or animals performed by any of the authors.

### Information Sources

Five databases (MEDLINE, CINAHL, Cochrane Central Register of Controlled Trials and Implementation Reports, PsycINFO, Web of Science) were initially accessed between January 1, 1992, and October 1, 2021. Subsequently, the search was extended through March 19, 2024.

### Eligibility Criteria

All studies, protocols, and intervention development papers published in peer-reviewed journals that reported and described interventions framed by the HAPA model for long-term conditions and targeted PA/exercise behavior, and written in English were eligible for inclusion. In order to provide the most current and detailed characteristics, we included protocols and intervention development papers as often authors publish each stage of intervention work separately. Studies and protocols lacking sufficient intervention reporting, including conference abstracts, non-peer-reviewed conference proceedings, editorials, unpublished theses and dissertations, letters, and editorial comments, were excluded.

### Search Strategy

We adopted a PICO framework (Additional file 1) including medical subject headings to identify published literature describing long-term conditions, physical activity/exercise, and HAPA model constructs in the design of interventions. To be as comprehensive and consistent as possible with terms related to each part of the PICO, we conducted an extensive MeSH term search prior to finalizing the search strategy. Papers were excluded if the HAPA model was not named in the paper or if multiple theories were used and the HAPA variable names were not clearly identified as part of the intervention. For the HAPA model search terms, we used “health action process approach”, “HAPA”, “HAPA model”, “HAPA approach”, and “HAPA theory” to be as inclusive as possible of varied terminology. A preliminary search strategy was piloted by two authors (ALS, SM) before a final search strategy was implemented for each database. We acknowledge global differences in terminology and categorization and we sought to conduct an expansive search by using a comprehensive and expanded list of MeSH terms (Additional file 2). For example, we included overweight/obesity as a population because the diagnoses are considered chronic conditions in some parts of the world. In addition, we reviewed reference lists and Google Scholar to identify articles associated with interventions and protocols.

### Data Extraction and Quality Assessment

A custom screening tool utilizing the PICO framework (Additional file 3) was applied by ALS to screen all titles and abstracts and determine papers meeting the inclusion criteria. The eligibility of full texts was assessed and documented by ALS and decisions independently checked by SM. Disagreements about study eligibility were resolved through discussion between authors. Two authors (ALS and SM) conducted the quality assessment (Table 1) using the JBI checklists. For seven interventions, there were two papers identified (protocol, RCT, or intervention development process description). In these cases, the RCT paper was used as the primary paper for intervention details unless the RCT paper deferred to a protocol paper for details.

**Table 1:** Quality assessment of randomized control trials and quasi-experimental studies

***Randomized control trials (associated protocol)***	Was true randomization used for assignment of participants to treatment groups?	Was allocation to treatment groups concealed?	Were treatment groups similar at the baseline?	Were participants blind to treatment assignment?	Were those delivering treatment blind to treatment assignment?	Were outcomes assessors blind to treatment assignment?	Were treatment groups treated identically other than the intervention of interest?	Was follow up complete and if not, were differences between groups in terms of their follow up adequately described and analyzed?	Were participants analyzed in the groups to which they were randomized?		Were outcomes measured in the same way for treatment groups?	Were outcomes measured in a reliable way?	Was appropriate statistical analysis used?	Was the trial design appropriate, and any deviations from the standard RCT design accounted for in the conduct and analysis of the trial?
Aliabad et al. [19]	√	√	√	√	?	?	√	√		√	√	√	√	√
Berli et al. [20] (Scholz et al. [41])	√	√	√	√	x	?	√	√		√	√	√	√	√
Daryabeygi-Khotbehsara et al. [21]	√	√	√	√	√	√	√	√		√	√	√	√	√
Döbler et al. [22]	√	√	√	x	x	?	√	√		√	√	√	√	√
Duan et al. [23]	?	?	√	?	?	?	√	√		√	√	√	√	√
Foulon et al. [24]	?	?	√	?	?	?	√	√	√		√	√	√	√
Greaves et al. [27]	√	√	x	√	√	√	√	√	√		√	√	√	√
Maxwell-Smith et al. [35, 28]	√	√	√	x	x	?	√	√	√		√	√	√	√
Hinrich et al. [29, 53]	?	√	N/A	√	N/A	√	√	N/A	N/A		N/A	N/A	N/A	√
Ho et al. [30]	√	√	√	x	x	√	√	√	√		√	√	√	√
Knoll et al. [31]	√	√	N/A	x	x	√	√	N/A	N/A		N/A	N/A	N/A	√
Ma et al. [33, 34]	√	√	x	x	x	√	√	√	√		√	√	√	√
O’Brien et al. [36]	√	√	N/A	x	x	√	√	N/A	N/A		N/A	N/A	N/A	√
Poppe et al. [38]	√	x	x	x	x	x	√	√	√		√	√	√	√
Reinwand et al. (Storm et al.) [39, 40]	√	√	x	√	?	√	√	√	√		√	?	√	√
Ströbl et al. [42]	√	√	√	x	x	?	√	?	√		√	√	√	√
Wilczynska et al. (Plotnikoff et al. ) [43, 44]	√	√	√	√	?	?	√	√	√		√	√	√	√

***Quasi-experimental studies***	Is it clear in the study what is the “cause” and what is the “effect” (i.e., there is no confusion about what variable comes first)?		Were the participants included in any comparisons	Were the participants included in any comparisons receiving similar treatment/care, other than the exposure or intervention of interest?		Was there a control group?	Were there multiple measurements of the outcome both pre and post the intervention/exposure?	Was follow up complete and if not, were differences between groups in terms of their follow up adequately described and analyzed?			Were the outcomes of participants included in any comparisons measured in the same way?		Were outcomes measured in a reliable way?	Was appropriate statistical analysis used?
Ghisi et al. (knowledge) [26]	√		√	x		√	√	√			√		√	√
Ghisi et al. (behavior) [25]	√		√	x		√	√	√			√		√	√
Platter et al. [37]	√		x	x		√	√	√			√		√	√

Joanna Briggs Institute critical appraisal checklists: Yes √; No x; Unclear ? Not applicable - N/A

The following protocols met PICO criteria for adult sample, chronic condition, use of HAPA theory in development of intervention: Scholz et al. [41], Hardcastle et al. [45], Hinrich et al. [53], Ho et al. [54], Karthijekan et al. [52], Liu et al. [32], Maxwell-Smith et al. [35], O’Brien et al. [36], Wilczynska et al. [43], Reinwand et al. [39], Smith et al. [57]

In keeping with the intent of the review to describe HAPA intervention details, two authors (ALS and CLH) extracted information about the main purpose of each paper, how HAPA variables were operationalized for each intervention (setting, mode of delivery, staff, and program length) using a customized Microsoft Excel spreadsheet (Microsoft Corporation, Washington DC, USA). We extracted the data from each paper that described which HAPA constructs were used and how the HAPA constructs were incorporated (operationalized) in each intervention. This information often was provided in tables that described intervention components. When a table was not included, we referred to the narrative description of the intervention to extract information about which HAPA variables were part of the intervention. The specific HAPA constructs had to be clearly named in the intervention description to be included as an intervention designed using the HAPA model. Further, we report the PA measures used and the effects of the interventions on physical activity outcome variables.

## Results

### Characteristics of Included Papers

**Fig. 2:**
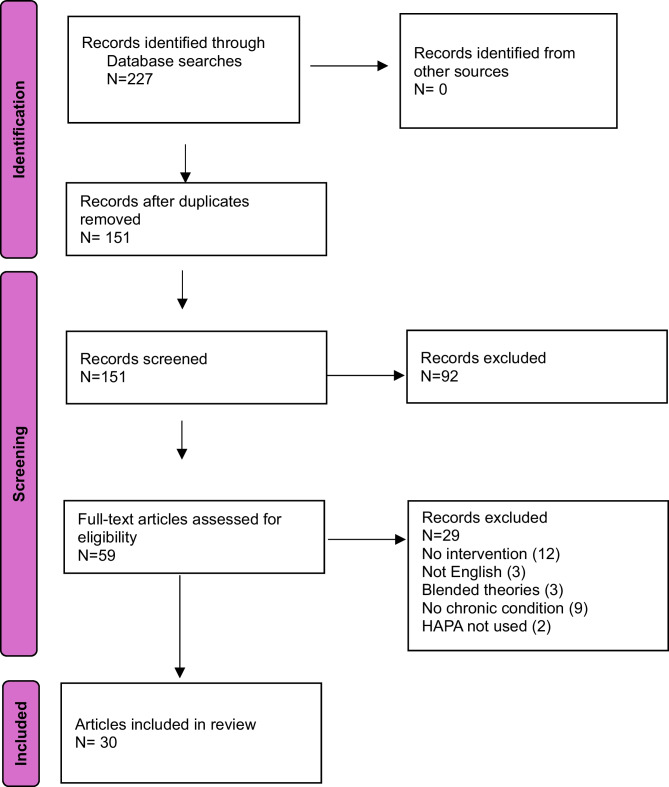
PRISMA-ScR flowchart

Our search identified 227 papers, of which 58 were duplicates and 18 were removed for other reasons (Fig. 2). Of the 151 papers screened for relevance, 59 were retained for full review. In the remaining 30 papers, 23 interventions were identified in 12 protocols [ 29, 31, 32, 35, 36, 39, 41, 43, 45, 52, 54, 57], three quasi-experiments [25, 26, 37], one intervention development paper [33], and 15 RCTs [19–24, 27, 28, 30, 34, 38, 40, 42, 44, 53].

The characteristics of the studies are presented in Table 2. Sample sizes of RCTs and quasi-experimental studies ranged from 28 to 790. The long-term conditions included coronary heart disease (CHD), type 2 diabetes mellitus (T2DM), cardiovascular (CV) risk factors, obesity, spinal cord injury (SCI), cancer, limited mobility/frailty, and osteoarthritis. Studies were conducted in 12 different countries between 2013 and 2020. The range of sample mean ages was 42–80 years and 12 studies included more males than females.

**Table 2:** Characteristics of HAPA studies

Authors (Ref no.)	Country	Main purpose	Sample size	Chronic condition	Age in years	Sex % Female
***Randomized control trials***						
Aliabad et al. [19]	Iran	Test effects of intervention with family support in maintenance of PA after discharge from rehabilitation	*N* = 96	CHD	Intervention *x̄* = 57.83 (SD = 8.71) Control *x̄* = 56.73 (SD = 9.0)	Intervention 16.7% Control 14.6%
Berli et al. Scholz et al. [20, 41]	Switzer-land	Test the effectiveness of a theory-based action control intervention using text messaging to promote daily physical activity	*N* = 121	Overweight Obesity	Intervention *x̄* = 48.33 (SD = 13.13) Control *x̄* = 43.97 (SD = 13.86)	Intervention 51.7% Control 50.8%
Daryabeygi-Khotbehsara et al. [21]	Iran	Test short-term effectiveness of intervention for compliance with diet and PA	*N* = 115	T2DM	Intervention *x̄* = 48.05 (SD = 5.96) Control *x̄* = 49.43 (SD = 6.15)	Intervention 30.5% Control 31.3%
Döbler et al. [22]	Germany	Test effectiveness of a telephone-delivered intervention to support health behavior modification	*N* = 199	T2DM	Intervention *x̄* = 51.6 (SD = 5.7) Control *x̄* = 52.2 (5.4)	Intervention 29.6% Control 29.7%
Duan et al. [23]	China	Test effects of web-based intervention to promote PA and fruit and vegetable consumption in pilot RCT	*N* = 83	CHD	Total Mean 49.18 (SD = 13.96)	Total 57%
Foulon et al. [24]	Canada	Test effectiveness of informational portrait vignettes for enhancing PA*	*N* = 79	SCI	*Motivation group:* Intervention *x̄* = Mean 44.06 (SD = 13.89); Control Mean 46.93 (SD = 6.97) *Volitional group:* Intervention *x̄* = 42.17 (SD = 10.77); Control Mean 44.61 (SD = 14.20)	*Motivation group:* Intervention 33.3% Control 50% *Volitional group*: Intervention 25% Control 34.8%
Greaves et al. [27]	UK	Test feasibility of intervention to increase weight loss, PA, and healthy diet	*N* = 108	High CV risk score	Intervention *x̄* = 66.6 (SD = 6.4) Control *x̄* = 63.7 (SD = 7.4)	Intervention 34.5% Control 26.4%
Hinrich et al. [53] Protocol Hinrich et al.	Switzerland	Test the effects of intervention to increase physical function, PA, quality of life, fall-related self-efficacy, and exercise self-efficacy	*N* = 209	Chronically ill, mobility limited, sedentary older adults	Intervention *x̄* = 79.6 (SD = 5.3) Control *x̄* = 80.1 (SD = 5.2)	Intervention 71.7% Control 75.7%
Ho et al. [30]	Hong Kong	Test feasibility of intervention to increase PA and reduce consumption of Western-pattern diet*	*N* = 223	Colorectal cancer	Diet + PA *x̄* = 63.2 (SD = 11.4) PA only *x̄* = 66.6 (SD = 9.5) Usual care *x̄* = 64.9 (SD = 9.4)	Diet + PA 33% PA only 29% Usual care 46%
Ma et al. [33, 34]	Canada	Test effects of intervention on PA levels, aerobic fitness, and psychosocial predictors of PA*	*N* = 28	Spinal cord injury	Intervention *x̄* = 45.79 (SD = 13.63) Control *x̄* = 45.57 (SD = 10.49)	Intervention 36% Control 43%
Maxwell-Smith et al. [28]	Australia	Test effectiveness of wearable technology coupled with action planning to increase PA	*N* = 68	Colorectal and endometrial cancer survivors at cardiovascular risk	Intervention group Mean 65.26 (SD = 7.41) Control group Mean 62.88 (SD = 8.37)	Intervention 61.8% Control 38.2%
Poppe et al. [38]	Belgium	Test short-term effects of intervention to alter levels of PA and sedentary behavior	*N* = 54	T2DM	Intervention PA *x̄* = 62.91 (SD = 7.16) Control *x̄* = 64.89 (SD = 8.62)	Intervention 29% Control 50%
Storm et al. [40]	Germany Netherlands	To test an 8-week web-based intervention to improve habit strength for PA and fruit and vegetable consumption among people who want to reduce their CV risk	*N* = 790	Cardiac rehabilitation	Intervention *x̄* = 50.9 (SD = 12.0) Control *x̄* = 50.8 (SD = 12.3)	Intervention 52.5% Control 47.5%
Ströbl et al. [42]	Germany	Test effects of intervention to increase PA in obesity rehabilitation	*N* = 467	Obesity	Intervention *x̄* = 48.54 (SD = 9.77) Control *x̄* = 48.03 (SD = 9.77)	Intervention 46% Control 44%
Plotnikoff et al. [44] Wilczynska et al. protocol [43]	Australia	Test an intervention to promote aerobic and resistance training among adults at risk of or diagnosed with T2DM	*N* = 84	Risk factors for T2DM or T2DM	Intervention *x̄* = 44.2 (SD = 13.5) Control *x̄* = 45.1 (SD = 14.7)	Intervention 71.4% Control 69.0%
***Quasi-experimental studies***						
Ghisi et al. (knowledge) [26]	Canada	Test whether the intervention results in more sustained knowledge, greater exercise behavior, and higher scores on HAPA constructs	*N* = 93	Outpatient cardiac rehabilitation	Intervention *x̄* = 67.35 (SD = 11.67) Control *x̄* = 67.42 (SD = 10.62)	Intervention 18.6% Control 30.0%
Ghisi et al. (behavior) [25]	Canada	Test the effect of education program on knowledge, HAPA constructs, and exercise behavior	*N* = 306	Outpatient cardiac rehabilitation	Intervention *x̄* = 64.30 (SD = 12.04) Control *x̄* = 63.58 (SD = 11.66)	Intervention 21.2% Control 23.1%
Platter et al. [37]	Austria	Test a brief health psychology intervention in acute care to increase PA behavior uptake	*N* = 193	CHD	Intervention *x̄* = 63.83 (SD = 8.9) Control *x̄* = 63.17 (SD = 9.1)	Intervention 29.6% Control 41%
***Protocols***						
Hardcastle et al. [45]	Australia	To increase moderate to vigorous PA among cancer survivors living remotely	n/a	Cancer	n/a	n/a
Hinrich et al. [29]	Germany	To test feasibility of a home-based exercise program for the elderly with structured support given by the general practitioner	n/a	Sedentary and frail older adults	n/a	n/a
Ho et al. [54]	Hong Kong	To test the acceptability and feasibility of a diet and physical activity intervention to prevent recurrence in colorectal cancer survivors	n/a	Colorectal cancer	n/a	n/a
Karthijekan and Cheng, [52]	Sri Lanka	To develop a culture-specific, motivated, and action-based intervention to improve PA level, exercise self-efficacy, and cardiovascular risk factors	n/a	CHD	n/a	n/a
Knoll et al. [31]	Germany	Evaluate the effects of intervention to increase uptake and maintenance of regular PA and to reduce symptoms of osteoarthritis	n/a	Osteoarthritis	n/a	n/a
Liu et al. [32]	Hong Kong	To reduce fatigue and frailty symptoms using individualized exercise with and without behavior change strategies	n/a	Frail older adults	n/a	n/a
Maxwell-Smith et al. [35] Protocol for Maxwell-Smith et al. RCT [28]	Australia	To test a low-intensity intervention increasing MVPA and reducing sedentary behavior	n/a	Endometrial and colorectal cancer with high CVD risk	n/a	n/a
O’Brien et al. [36]	New Zealand	Test effectiveness of intervention to improve long-term PA and functional abilities	n/a	Osteoarthritis of hip and knee	n/a	n/a
Reinwand et al. [39] Protocol for Storm et al. [40]	Netherlands	To increase PA and fruit and vegetable consumption following discharge from cardiac rehabilitation	n/a	Cardiac rehabilitation	n/a	n/a
Scholz et al. [41] [Protocol for Berli et al. [20]	Switzer-land	Test the effectiveness of intervention to promote daily PA in overweight or obese adults	n/a	Overweight/obesity and sedentary	n/a	n/a
Smith et al. [57]	Australia	To develop and test a HAPA-based intervention to initiate and maintain stroke secondary prevention behaviors	n/a	Stroke	n/a	n/a
Wilczynska et al. [43] Protocol for Plotnikoff et al. [44]	Australia	Test an intervention to promote aerobic and resistance training among adults at risk of or diagnosed with T2DM	n/a	Risk factors for T2DM or T2DM	n/a	n/a

*n/a* not applicable, * used HAPA stage in intervention, *PA *physical activity, *CVD* cardiovascular disease, *CHD *coronary heart disease, *T2DM* Type 2 Diabetes Mellitus

### Operationalization of HAPA Constructs

The studies operationalized 23 PA interventions in various venues and modes of delivery (Table 3). Interventions were delivered in outpatient departments (*n* = 7), rehabilitation centers (*n* = 1), community venues (*n* = 4), research facilities (*n* = 2), acute wards (*n* = 2), in home (*n* = 2), primary care office (*n* = 1), telephone or text messages (*n* = 9), online/app (*n* = 7), and 12 used more than one setting. The modes of intervention content delivery (multiple for some interventions) were face-to-face in person (*n* = 16), telephone (*n* = 5), internet (*n* = 7), or cellular phone texts/app (*n* = 2), or a combination of these modes (*n* = 12).

**Table 3:** HAPA interventions

Author, year	Delivery venue	Staff	Intervention length	No. of sessions	Delivery mode	Description of delivery content
Aliabad et al. (2014) [19]	Outpatient department	Not specified	Not specified but 4-month follow-up	3	Face-to-face individual sessions	**Session 1:** Discussion (PA risk perception, outcome expectancies and task-self efficacy). Booklet (information about PA and health in cardiac patients, PA benefits, persuasive messages, and reminders of successful experiences during CR, and ambiguity removal) **Session 2:** Discussion (overcoming barriers to PA and goal setting). Booklet (completion of PA action plan, plans to overcome barriers and goal setting, journal of feelings after PA. Examples of how to overcome barriers given) **Session 3:** Inclusion of social support person. Discussion (social support strategies—sharing goals, involvement of significant others in PA). Booklet (HAPA-based educational booklet given to participants at end)
Berli et al. (2016) [20] Scholz et al. (2014) [41]	Home-based; cellphone	Trained study staff	14 days	28 texts	Text messages	**Baseline:** Overweight/obese couples given information leaflet containing information on PA benefits for health and weight management. Quiz, with feedback on incorrect answers **Dyadic action control group:** couples collaboratively set activity goals supervised by study staff **Days 1–14:** 2 × short, standardized text messages on weekdays morning and afternoon sent from participant’s partner. Messages related to PA self-monitoring, behavioral intentions, and goals **Individual action control group:** Individuals set activity goals supervised by study staff **Days 1–14:** 2 × short, standardized text messages on weekdays morning and afternoon sent from study staff. Messages related to PA self-monitoring, behavioral intentions, and goals **Potential confounders:** Participants asked to wear accelerometers and keep activity diaries for 28 days (including the 14-day intervention period)
Daryabeygi-Khotbehsara et al. (2021) [21]	Outpatient department	Nutritionist	4 weeks	4	Face-to-face 2-h group classes	Video about diabetic risks **Week 1**: Low-fat food consumption **Week 2:** Carbohydrate counting **Week 3:** PA **Week 4:** Summary session **Group-based discussions:** (10–12 people) advantages and disadvantages, social support and tackling disapproval, and barriers and facilitators to target behavior **Planning:** Goal setting, how to deal with setbacks and difficult situations
Döbler et al. (2018) [22]	Rehabilitation center and telephone	Counselor	12 months	13	Baseline 1 h in-person MI session. Monthly telephone counselling	Five intervention modules: (1) medication adherence, (2) PA, (3) diet, (4) smoking cessation, (5) emotional problems **MI session:** Developing individualized action plan and coping plans (identifying barriers, problem solving, soliciting social support, and feedback on performance) using 2-stage HAPA for 1–2 behaviors chosen by participant **Telephone support:** Assessment of emotional problems. Discussion, evaluation, and extension of personal plans
Duan et al. (2018) [23]	Online	Nurse reminders prior to sessions	8 weeks	8	Internet-based course 1 × per week	PA (weeks 1–4) and fruit and vegetable consumption (weeks 5–8) **Weeks 1 and 5:** Risk perception, outcome expectancies, goal-setting activities **Weeks 2 and 6:** Development of action plans **Weeks 3 and 7:** Revision and adjustment of previous action plans, development of coping plans **Weeks 4 and 8:** Revision and adjustment of previous coping plans and development of behavior-specific social support. Self-efficacy included in all weeks. Participants contacted by CR nurse prior to each session by telephone as a reminder
Foulon et al. (2013) [24]	Online	PA vignette	1 session	1	Online asynchronous individual session	Vignettes matched to sex, age, mode of mobility and SCI level. 7 days after baseline demographics and staging assessment, link emailed with vignette assigned by stage or control **Participants assessed in the motivational phase** viewed a tailored vignette focused on risk perception, outcome expectations, task self-efficacy and intention formation for PA **Participants assessed in volitional phase** viewed a tailored vignette focused on action and coping planning, action control, and maintenance and recovery self-efficacy for PA **Control vignette** non-tailored biography of man with SCI, including traumatic incident and how he helped others in the following years. No mention of PA
Ghisi et al. (2015a) [25]; Ghisi et al. (2015b) [26]	Outpatient department and community venue	Multi-disciplinary team	6 months	24	Face-to-face 30-min education session prior to exercise with large and small groups	Lecture, workbook, online videos, and pamphlet Topics covered: exercise safety, nutrition, risk management, medications, stress management, and lifestyle. Sessions contained: 1. Educational content 2. Learning activities 3. Learning assessments 4. Behavioral-based action planning 5. Assessment of motivation 6. Confidence to incorporate change into lifestyle
Greaves et al. (2015) [27]	Community venues	2 lifestyle coaches	9 months	9	2-h group sessions weekly × 4 then 1.5-h support sessions × 5	**Group sessions weeks 1–4:** (10–12 people) targeting PA and healthy eating. Developing motivation, understanding behavior change, increasing risk perception, social support, assessing PA levels and diet, action planning, goal setting, coping planning, social support, action planning and updates, outcome expectancies, goal setting, social support **Handbook** with weekly “take away” tasks and information **Support sessions 1.5, 2, 4, 6, and 9 months:** supporting maintenance via self-regulatory cycles of reflection/feedback, self-monitoring, relapse prevention techniques, revising action plans
Hardcastle et al. (2019) [45]	Skype or FaceTime	Health coaches	12-week	4–6	1-h session then 30-min sessions weeks 2, 4, 8; optional sessions weeks 6, 10	**Session 1, week 1:** Review technical issues and features of the Fitbit; importance of MVPA and fostering positive outcome expectancies and confidence towards PA; guidance to create PA action plans for the following 3 weeks and to begin self-monitoring activity **Sessions for weeks 2, 4, 8:** Provide support, problem solving and assist to revise goals and action plans as they progress **Sessions optional for weeks 6, 10:** Health-coaching sessions negotiated between the health coach and the participant based on both data from the Fitbit dashboard concerning progress and participants’ perceptions concerning support needs
Hinrich et al. (2009) [29] (2016) [53]	Primary care and telephone	Exercise therapist physiologist/instructor/PT	12 weeks	8	Face-to-face primary care, telephone, and home-based exercise	Multidimensional (strength, endurance, balance, flexibility) home-based exercise programme with consultations including personal attention, instruction, and methods fostering behavior change (goal setting, action planning, dealing with barriers). Pedometers and resistance bands provided **Weeks 1, 2, 4, 7, 11:** Face-to-face consultation in primary care setting **Weeks 5, 8, 12:** Telephone consultations **Workbook** on topics from 8 intervention sessions, worksheets for goal setting, and activity logs
Ho et al. (2020) [30]	Outpatient department and telephone	Trained study staff	12 months	28	Face-to-face baseline visit, 24 telephone calls, 4 group meetings	PA intervention group: **Week 1:** Individual face-to-face motivational interview (PA benefits, exploring PA options, goal setting, barriers and facilitators to PA, demonstration of pedometer use) **Biweekly:** Motivational telephone calls (progress monitoring, encouragement and reinforcement, problem solving) **Quarterly:** Group meeting for social support, mailed newsletters sharing participant experiences **Monthly:** HAPA-stage-of-change matched information pamphlets by mail
Karthijekan et al. (2022) [52]	Outpatient cardiology clinic and telephone	Registered Nurse	12 weeks	10	Face-to-face baseline visit: 3 monthly face-to-face education sessions, 3 monthly face-to-face group exercise, 3 telephone calls	**Individual preparatory phase:** 60-min face-to-face meeting for goal setting and action planning **Week 1**: educational booklet culturally relevant written resources and face-to-face group education and learning exercises **Week 3**: Telephone counseling **Week 5**: Face-to-face group education and exercise **Week 7**: Telephone counseling **Week 9**: Face-to-face group education and exercise **Week 11**: Telephone counseling
Knoll et al. (2018) [31]	Outpatient department and telephone	Trained study staff	12 months	5	Computer-assisted face-to-face and telephone support	**Brief motivational 6-page printed leaflet:** Information on increasing PA for persons with knee osteoarthritis, different intensities of PA and PA guidelines for persons with knee osteoarthritis, and addresses outcome expectancies, risk perceptions, self-efficacy beliefs, and role models related to PA. Crossword quiz **Week 1:** 1-h computer-assisted face-to-face (seated next to staff and computer); 4 sections focused on outcome expectancy, mastery/self-efficacy, goal setting, planning **Weeks 3, 27, 50, 52:** 4 computer-assisted phone-based “booster” interventions focused on planning, self-efficacy, action control for PA **Weeks 1–4, 25–28, 50–53:** completion of activity calendars
Liu et al. (2019) [32]	Community venue	Behavior change specialist/exercise instructor/health talk deliverer	16 weeks	19	Face-to-face	**Weeks 1–3:** 60-min face-to-face behavior change sessions (goal initiation and planning) **Weeks 4, 8, and 12:** 60-min face-to-face behavior change sessions (action execution—experiences of success, social persuasion through sensible feedback and encouragement, peer sharing, positive physiological and emotional responses to regular exercise) **Weeks 4–16:** 1 × per week 45–60-min exercise sessions (balance training, resistance exercises, and aerobics training) (action execution phase). Expected to do home-based exercise 3 × per week 30–45 min **Booster sessions:** 2 months and 6 months after program (unspecified)
Ma et al. (2019) [34], (2020) [33]	Research facility or telephone	Exercise therapist physiologist/instructor /PT	8 weeks	9	Face-to-face or Skype or telephone	**Baseline:** 1-h face-to-face session (current PA levels, goal setting, action planning, identification of barriers and potential solutions, resources identified, and demonstration of PA) **Weeks 1–8:** 1 × per week PA coaching sessions lasting 10–15 min (monitor PA and feedback, goal setting and problem solving if required, education about PA, information on local resources, tailored PA prescription) Tailoring and the individual’s HAPA stage used throughout to match BCT strategies to participant needs and preferences
Maxwell-Smith et al. (2019) [28]	Outpatient department and telephone	Behavior change specialist	8 weeks	3	Fitbit Alta™, 2 group sessions and one telephone call	3 health-coaching intervention components **Fitbit Alta** provided as motivating strategy **Week 1 group session:** Risk perception, outcome expectancy, action SE and intention, Fitbit™ fitting and instruction **Week 4 group session:** Coping planning, maintenance SE **Week 8 telephone call:** Feedback and support session focused on self-regulation, maintenance self-efficacy, and coping planning **Printed booklet** on PA guidelines, strength exercises, benefits of PA, logs, confidence building, barrier solving, coping planning, action-planning, and goal setting activities
O’Brien et al. (2018) [36]	Research facility	Exercise therapist physiologist /instruct-or/ PT	12 weeks	29	Face-to-face	Program individualized based on stage at entry **Weeks 1, 2, 3, 6, 12:** Counselling sessions (exercise benefits, building confidence, creating plans and habits, staying in control, visualising the future) **Weeks 1–12:** 2 × per week 60-min progressive group exercise class (aerobic, strength, and flexibility components) (maximum 5 people). Stage-adapted and participants encouraged to exercise within their capabilities. Home-based exercises prescribed by exercise physiologist **Workbook**: On benefits of PA for people with osteoarthritis, setting goals, forming habits, and making plans. Reflective activities to complete at home
Platter et al. (2016) [37]	Acute hospital ward	Nurse	1 session	1	Face-to-face	1-h group education session just prior to discharge in 3 parts: **Part 1:** Information about PA for cardiac patients with opportunity for questions **Part 2:** Development of personal action and coping plans for PA (when, where, and how) **Part 3:** Group discussion involving sharing of personal plans
Poppe et al. (2019) [38]	Online	Researcher contacts if not engaged	5 weeks	5	Website with optional app-based support	**Weeks 1–5:** 1 × per week online sessions to create and evaluate personal goals to reduce SB or increase PA. Action plan generated based on answers to questions about current PA, specific plan to increase PA, potential barriers selected, possible solutions, and decisions about self-monitoring. Email contact by researcher if did not attend weekly session, followed by a phone call if no response **Daily:** Optional mHealth app providing daily support (prompting self-monitoring, goal review and action planning, barrier identification, problem solving, gamification via quizzes to provide information on consequences and points collection)
Reinwand et al. (2013) [39] Storm et al. (2016) [40]	Online	Not applicable	8 weeks	8	Website	1 × per week online intervention after discharge from CR PA (weeks 1–4) and fruit and vegetable consumption (weeks 5–8) **Weeks 1 and 5:** Questionnaire and personalized feedback, increase risk perception, outcome expectancies, defining own health outcomes **Weeks 2 and 6:** Personalized feedback, development of action plans **Weeks 3 and 7:** Personalized feedback, self-reflection, revision and adjustment of action plans, defining personal barriers, development of coping plans **Weeks 4 and 8:** Personalized feedback, adjustment of coping plans, and development of behavior-specific social support Participants sent autogenerated weekly email reminders to take part in sessions
Smith et al. (2023) [57]	Community	Group facilitator with experience and training	8 weeks	5	In person and telehealth	**8-week online or in-person based on preference** **Session 1:** One-on-one with facilitator online; introduction/behavior change; assigned to small group **Sessions 2, 3, 4**: Group; 1 week apart; HAPA constructs (motivation, planning, monitoring) **Sessions 4 and 5**: Group; spaced 4 weeks apart; HAPA construct-toolkit to make changes
Ströbl et al. (2013) [42]	Acute setting and home-based; telephone	Rehabilitation staff/sports therapist	6 months	8	Face-to-face group sessions and telephone	**3-week multimodal, structured, interdisciplinary standard treatment pre-discharge** (complete medical check, CVD risk factor assessment, daily nutrition advice, cooking seminars, daily PA sessions, 2 × psychoeducation seminars) **Group-based counselling session** 50 min, 4–10 people (self-reflection, self-monitoring, action planning, goal setting, barriers and facilitators, coping planning). Booklets for plans **Individual counselling session** (10 min) prior to discharge (review action and coping plans) **Weeks 2, 5, 9, 13, 24 post inpatient discharge:** Telephone calls (5–10 min) (encouragement, review goals, feedback on performance, follow-up prompts, relapse prevention)
Wilczynska et al. (2016) [43]	Community venues and app-based	Clinical psychologist	20 weeks	5	Face-to-face and app-based	**Weeks 1–10:** 5 × 90-min face-to-face group sessions (30 min for cognitive mentoring and group training incorporated self-efficacy, action planning, and coping planning strategies, 60 min for outdoor exercise session) **Weeks 1–20:** Smartphone app tailored to geographic location for goal setting, self-monitoring, outdoor physical environment, challenges, and links to social media

Replicability score based on details provided in papers: 3 replicable, 2 marginally replicable, 1 not replicable

*CR* cardiac rehabilitation, *CVD* cardiovascular disease, *PA* physical activity, *SCI* spinal cord injury

The range of HAPA constructs included in interventions was 3 to 9 variables (Table 4). The three most frequently operationalized HAPA variables were *action planning*, *coping planning*, and *intention.* The HAPA variable least likely to be implemented was *recovery self-efficacy*. Only three papers [24, 30, 33] reported using the stage approach to the HAPA model to design and deliver the intervention by identifying participants as pre-intenders, intenders, or actors based on pre-assessment.

**Table 4:** Number of HAPA variables included

Author, year	Action control	Action planning	Coping planning	Goal setting	Intention	Maintenance self-efficacy	Outcome expectancy	Risk perception	Recovery self-efficacy	Task self-efficacy
Aliabad et al. (2014) [19]	─	√	√	─	√	√	√	√	√	√
Berli et al. (2016) [20]	√	√	─	√	√	─	─	─	─	─
Daryabeygi-Khotbehsara et al. (2021) [21]	─	─	√	√	√	─	─	√	─	─
Döbler et al. (2018) [22]	─	√	√	─	√	√	─	─	─	√
Duan et al. (2018) [23]	─	√	√	√	√	─	√	√	─	√
Foulon et al. (2013) [24]	√	√	√	─	√	√	√	√	√	√
Ghisi et al. (2015) × 2 [25, 26]	─	√	√	─	√	√	√	√	─	√
Greaves et al. (2015) [27]	─	√	√	√	─	─	√	√	─	√
Hardcastle et al. (2019) [45]	─	√	─	─	√	√	√	√	─	√
Hinrich et al. (2011), (2016) [53]	√	√	√	√	√	√	√	─	─	√
Ho et al. (2013), (2020) [30, 50]	√	√	√	√	√	√	√	─	√	─
Karthijekan et al. (2022) [52]	√	√	√	√	√	√	√	√	─	√
Knoll et al. (2018) [31]	─	√	√	√	─	─	√	─	─	√
Liu et al. (2019) [32]	√	√	√	√	√	─	√	√	─	√
Ma et al. (2019), (2020) [33, 34]	─	√	√	√	√	√	√	√	√	√
Maxwell-Smith et al. (2018) [35]	─	√	√	√	√	─	√	√	─	√
O’Brien et al. (2018) [36]	√	√	√	─	√	√	√	√	√	√
Platter et al. (2016) [37]	√	√	√	─	√	─	─	─	─	─
Poppe et al. (2019) [38]	─	√	√	√	─	─	─	─	─	─
Reinwand et al. (2013) [39] and Storm et al. (2016) [40]	─	√	√	√	√	√	√	√	─	√
Smith et al. (2023) [57]	√	√	─	─	√	─	√	√	─	√
Ströbl et al. (2013) [42]	√	√	√	√	√	─	─	─	─	√
Wilczynska et al. (2016) [43]	─	√	√	─	√	─	√	√	─	√

**Table 5:** Physical activity instruments and outcomes

**Author**	**PA measure**	**Baseline measures**			**Post-test measures**			**Significance**
**Randomized controlled trial**								
Aliabad et al. [19]	*Godin Leisure-time Exercise Questionnaire modified to give exercise total score* (ETS) *Bruce protocol treadmill test* (ml/kg/min)	CG mean ± SD 81.3 (± 58.9) 43.56 (± 6.6)	IG mean ± SD 70.1 (± 62.1) 43.4 (± 6.7)		CG mean ± SD 47.4 (± 59.4) 36.7 (± 6.8)	IG mean ± SD 182.9 (± 110.2) 42.5 (± 6.4)		Between-group differences *p* < 0.001 *p* < 0.001
Berli et al. [20]	*Accelerometer wear (28 days)* % achieving 30 min of MVPA/day performed in 10-min bouts MVPA (min/day)	CG 21.0% of days (SD ± 18.3, range = 0 to 81) across 28-day period Median 40.0 (IQR not reported) Probability of achieving recommended daily MVPA (23.0%; 95% CI 17.4–30.6%)			IG 32.7% of days (SD ± 22.6, range = 0 to 78) across 28-day period Median 45.0 (IQR not reported) Probability of achieving recommended daily MVPA (36.5%; 95% CI 28.4–45.5%)			No significant effect on overall daily moderate–vigorous PA in minutes *p* = 0.623
Döbler et al. [22]	*PA self-report Likert measure for winter and summer, summed for total exercise index*	Not reported			CG: 10% increase in exercise index	IG 26% increase in exercise index		*p* < 0.001
Duan et al. [23]	*Chinese short version of International Physical Activity Questionnaire* Total PA score (minutes/week)	CG: Mean 157.4	IG Mean 143.4		CG: Mean 136.2	IG Mean 218.3		Interaction time × PA *F* (df), *η*^2^ 9.25 (1,81), .10, *p* < 0.003
Greaves et al. [27]	*Accelerometer wear* MVPA (min/day) Steps per day, validated wear time (counts/min) MVPA (min/day) Steps per day, validated wear time (counts/min)	CG: 21.6 (16.5) 6551 (2499) 270.5 (106.6)	IG 22.5 (22.3) 6420 (3016) 240.6 (118.4)		CG: 0–4 months change − 0.92 (3.44) 309.1 (1932) 22.5 (72.3) 0–12 months change 3.27 (18.82) 166.1 (2291) 19.0 (96.2)	IG 0–4 months change 1.79 (14.10) 126.4 (1400) 15.0 (74.0) 0–12 months change − 0.51 (14.88) − 141.0 (1903) 1.36 (74.52)		Between-group differences *p* = 0.587 *p* = 0.535 *p* = 0.457 *p* = 0.251 *p* = 0.367 *p* = 0.116
Hinrich et al. [53]	*Range of functional tests* Chair-rise test, (s) Timed up and go (s) Hand grip strength (kg) Chair sit-and-reach left leg (cm) Chair sit-and-reach right leg (cm) Tandem stand (s) Tandem walk (steps) 2-min step in place (cycles) Pedometer (steps/day)	CG Mean ± SD 23.1 ± 16.1 14.0 ± 5.8 23.1 ± 16.1 11.9 ± 12.7 12.1 ± 12.5 8.4 ± 2.7 2.7 ± 3.0 44.7 ± 13.7 2989 ± 1677	IG Mean ± SD 19.5 ± 6.2 13.4 ± 5.3 19.5 ± 6.2 15.0 ± 11.9 15.0 ± 12.1 8.6 ± 2.3 2.9 ± 2.8 43.8 ± 13.2 3602 ± 2022		CG Adjusted post-intervention mean (95% CI) 19.0 (17.8–20.3) 13.8 (13.1–14.4) 24.4 (23.8–25.1) − 10.7 (− 12.1 to − 9.3) − 10.3 (− 11.7 to − 8.9) 8.3 (8.0–8.7) 3.1 (2.7–3.5) 45.9 (44.1–47.7) 3403 (3091–3715)	IG Adjusted post-intervention mean (95% CI) 19.0 (17.8–20.2) 13.1 (12.5–13.7) 24.6 (24.0–25.3) − 10.0 (− 11.6 to − 8.5) − 10.2 (− 11.7 to − 8.7) 8.8 (8.4–9.1) 3.4 (3.0–3.8) 47.7 (45.7–49.7) 3196 (2841–3551)		Difference (IG-CG) Between adjusted post-intervention Mean (95% CI); *p* − 0.1 (− 1.8 to 1.7); = 0.94 − 0.7 (− 1.5 to − 0.2); = 0.13 0.2 (− 0.7 to 1.1); = 0.66 0.6 (− 1.4 to 2.7); = 0.54 0.1 (− 1.9 to 2.2); = 0.92 0.4 (− 0.1 to 1.0); = 0.09 0.3 (− 0.3 to 0.9); = 0.32 1.8 (− 0.9 to 4.5); = 0.19 − 207 (− 618 to 203); = 0.32
Ma et al. [33]	*Leisure time PA questionnaire* Total LTPA (min per week) MV LTPA (min per week) *Accelerometer* total counts *Graded exercise test on arm ergometer* Absolute VO_2peak_ (l/min) Relative VO_2peak_ (ml/kg/min) Peak aerobic work rate (watts)	CG Mean ± SD (95% CI) 274 ± 300 (136–414) 83 ± 67 (49–117) 8.48 × 10^5^ ± 7.59 × 10^5^ 1.13 ± 0.46 (0.89–1.37) 15.00 ± 5.72 (12.11–17.88) 70 ± 37 (51–88)	IG Mean ± SD (95% CI) 212 ± 195 (73–351) 68 ± 57 (34–102) 5.62 × 10^5^ ± 1.88 × 10^5^ 1.16 ± 0.38 (0.92–1.40) 15.94 ± 4.24 (13.05–18.83) 82 ± 27 (63–100)		CG Mean ± SD (95% CI) 147 ± 192 (− 13 to 307) 48 ± 69 (− 75 to 171) 5.98 × 10^5^ ± 3.90 × 10^5^ 1.06 ± 0.40 (0.82–1.29) 13.94 ± 5.51 (10.96–16.93) 65 ± 36 (46–84)	IG Mean ± SD (95% CI) 405 ± 364 (245–564) 290 ± 319 (96–483) 7.02 × 10^5^ ± 2.67 × 10^5^ 1.30 ± 0.43 (1.06–1.54) 17.83 ± 4.91 (14.84–20.81) 87 ± 30 (68–106)		Group × time interaction *F*, *p*, Cohen’s *d* 11.07, 0.003, 0.89 10.79, 0.003, 1.04 4.51, 0.044, 0.31 13.80, 0.001, 0.58 25.37, < 0.001, 0.94 17.56, < 0.001, 0.68
Maxwell et al. [28]	Accelerometer (min per week) MVPA (min/week) Moderate PA (min/week) MV10 (completing ≥ 150 min/week)	CG (mean and CI) 261 (202, 337) 247 (192, 317) *N* = 5 (15%)	IG (mean and CI) 267 (207, 344) 254 (198, 325) *N* = 6 (18%)		CG (mean and CI) 240 (185, 311) 217 (168, 280) *N* = 7 (21%)	IG (mean and CI) 312 (242, 402) 295 (230, 379) *N* = 9 (27%)		Group × Time *F*_1,126_ 5.14, *p* = 0.025 6.77 ns 0.00 ns
Poppe et al. [38]	*International Physical Activity Questionnaire* Total PA (min/day) MVPA (min/day) *Accelerometer wear (10 days)* Total PA (min/day) MVPA (min/day) Daily steps *International Physical Activity Questionnaire* Total PA (min/day) MVPA (min/day) *Accelerometer wear (10 days)* Total PA (min/day) MVPA (min/day) Daily steps	CG Mean ± SD RCT 1 86.1 ± 70.5 17.9 ± 24.4 262.5 ± 78.6 23.2 ± 12.9 6203.1 ± 2284.4 RCT 2 134.6 ± 96.1 65.2 ± 73.7 335.4 ± 91.7 29.3 ± 22.1 7929.7 ± 2976.1	SB IG Mean ± SD RCT 1 138.0 ± 99.9 47.3 ± 67.2 239.1 ± 48.8 20.2 ± 13.9 6083.9 ± 1343.30 RCT 2 98.4 ± 94.3 36.1 ± 39.6 291.2 ± 66.5 29.0 ± 23.4 7809.2 ± 3231.2	PA IG Mean ± SD RCT 1 113.8 ± 90.5 53.2 ± 72.0 255.2 ± 69.0 17.1 ± 15.7 5364.4 ± 2219.3 RCT 2 109.4 ± 108.0 63.9 ± 81.8 362.4 ± 82.8 25.0 ± 12.3 8271.7 ± 2464.3	CG Mean ± SD RCT 1 124.5 ± 96.0 62.0 ± 75.8 267.2 ± 83.1 19.4 ± 14.8 6292.1 ± 2480.4 RCT 2 117.6 ± 86.0 64.3 ± 75.2 340.3 ± 73.8 23.5 ± 14.8 7779.8 ± 2147.9	SB IG Mean ± SD RCT 1 150.1 ± 100.4 63.6 ± 76.9 235.1 ± 33.9 19.4 ± 13.8 6001.0 ± 1107.3 RCT 2 104.2 ± 48.8 60.6 ± 38.7 301.3 ± 73.2 30.9 ± 17.6 8479.7 ± 3343.1	PA IG Mean ± SD RCT 1 143.8 ± 91.5 48.7 ± 41.4 270.0 ± 78.3 25.50 ± 15.8 6549.7 ± 2313.7 RCT 2 168.9 ± 99.5 106.4 ± 78.4 366.0 ± 87.6 17.2 ± 10.5 7663.5 ± 2797.7	No significant differences PA intervention group within-group difference MVPA* p* = 0.049 < 0.01 (between-group difference PA vs CG for total PA) No significant differences
Ströbl et al. [42]	*Freiburg Questionnaire for PA* PA duration (h/week) PA energy expenditure (kcal/week) PA duration (h/week) PA energy expenditure (kcal/week)	CG Mean ± SD 6.5 ± 5.1 2829.6 ± 2315.5	IG Mean ± SD 5.6 ± 4.9 2398.4 ± 2161.0		CG Mean ± SD (6 months) 8.5 ± 5.2 3451.3 ± 2115.4 (12 months) 7.45 (5.03) 3196.6 ± 2251.7	IG Mean ± SD (6 months) 9.2 ± 6.1 3654.0 ± 2517.6 (12 months) 7.8 ± 5.6 3384.4 ± 2574.7		*p* = 0.014, *η*^2^ 0.014 *p* = 0.009, *η*^2^ 0.009 *p* = 0.0118, *η*^2^ 0.014 *p* = 0.008, *η*^2^ 0.015
Plotnikoff et al. [44]	*Functional tests* The single-stage treadmill walking test (ml/kg/min) Timed up and go (s) Arm curls in 30 s (reps) Chair stand test (reps) *Pedometer wear (7 days)* PA (steps per day) *Functional tests* The single-stage treadmill walking test (ml/kg/min) Timed up and go (s) Arm curls in 30 s (reps) Chair stand test (reps) *Pedometer wear (7 days)* PA (steps per day)	CG Mean ± SD 33.6 ± 8.2 9.1 ± 2.1 12.4 ± 4.0 10.3 ± 2.4 6117 ± 3203	IG Mean ± SD 30.5 ± 8.2 8.6 ± 1.5 11.0 ± 2.7 10.1 ± 1.9 6799 ± 3730		CG Change from baseline Mean (95% CI) (10 weeks) − 1.3 (− 3.6, 0.9) − 0.6 (− 1.0, − 0.2) 2.6 (1.9, 3.4) − 1.7 (− 2.2, − 1.1) − 160 (− 1040, 720) (20 weeks) − 0.2 (− 2.3, 1.8) 0.8 (0.5, 1.2) − 2.0 (− 2.9, − 1.1) − 1.2 (1.9, − 0.5) 720 (− 543, 1983)	IG Change from baseline Mean (95% CI) (10 weeks) 3.1 (0.9, 5.4) 1.1 (0.7, 1.6) − 2.4 (− 3.1, − 1.6) 1.8 (1.2, 2.3) 1170 (254–2086) (20 weeks) 2.6 (0.5, 4.7) − 0.9 (− 1.2, − 0.5) 3.1 (2.1, 4.0) 2.7 (2.0, 3.4) 1449 (115, 2782)		Between-group differences *p*, Cohen’s *d* (10 weeks) = 0.007, 0.68 < 0.000, − 1.16 < 0.000, 1.46 < 0.000, 1.45 = 0.043, 0.67 (20 weeks) = 0.062, 0.43 < 0.000, − 1.21 < 0.000, 1.36 < 0.000, 1.37 = 0.073, 0.56
**Quasi-experimental studies**								
Ghisi et al. [26]	*Two self-report questions:* Weekly PA (h) (mean ± SD) Walking 3–4 times per week or more, *n* (%)	CG 5.64 ± 6.6 48 (96.0%)	IG 5.9 ± 5.3 43 (100.0%)		CG Post-CR 7.4 ± 6.4 6 months post-CR 6.1 ± 4.7 Post-CR 50 (100.0%) 6 months post-CR 6.1 ± 4.7 50 (100.0%)	IG Post-CR 6.9 ± 4.5 6 months post-CR 9.1 ± 6.8 Post-CR 43 (100.0%) 6 months post-CR 43 (100.0%)		Significant within groups change in self-reported weekly physical exercise hours Pre vs post CR CG* p* < 0.05 Pre vs 6-month post CR IG *p* < 0.001
Ghisi et al. [25]	*Two self-report questions:* Weekly PA (h) (mean ± SD) Walking 3–4 times per week or more, *n* (%)	CG 5.3 ± 7.0 104 (67.5%)	IG 4.6 ± 4.5 100 (69.9%)		CG 7.5 ± 6.0 82 (89.1%)	IG 8.7 ± 13.4 65 (81.3%)		Within groups change in self-reported weekly PA CG *p* ≤ 0.05 IG *p* ≤ 0.001 Significant within groups change in self-reported walking 3–4 times per week or more CG *p* ≤ 0.05 IG *p* ≤ 0.01 Between-group differences for pre to 6 month post-CR
Platter et al. [37]	*Adapted Kaiser PA Survey* PA (min/week) Mean ± SD	CG 99.0 ± 160.2	IG 131.8 ± 150.1		CG change in PA 2 months − 3.5 ± 207.4 6 months + 46.6 ± 154.2	IG change in PA 2 months + 80.4 ± 213.2 6 months + 51.9 ± 180.4		Significant within groups change in self-reported weekly PA at 6 months CG *p* 0.008 IG *p* = 0.012 No between-group differences

RCTs without PA measures [21, 24, 30] excluded from table

*CG* control group, *IG* intervention group, *IQR* interquartile range, *MVPA* moderate to vigorous physical activity, *PA* physical activity, *SD* standard deviation

### Physical Activity Measures and Outcomes

Fourteen of the RCT and quasi-experimental studies reported one or more measures that allowed for change in PA to be assessed: self-reported PA (*n* = 9) [19, 22, 23, 25, 26, 33, 37, 38, 42], accelerometer wear (*n* = 4) [20, 27, 28, 33, 38], pedometer (*n* = 3) [29, 44, 53], aerobic fitness via treadmill/arm ergometer test (*n* = 3) [19, 33, 44], Timed Up and Go (*n* = 2) [44, 45], chair rise (*n* = 2) [44, 45]. Seven studies used self-report measures as the sole measure of PA [22, 23, 25, 26, 37, 42]. However, three of these did not use validated self-report measures [22, 25, 26] (Table 4). Seven RCTs reported significant PA differences in favor of the intervention in at least one measure [19, 22, 23, 33, 38, 42, 44], although these were not always apparent for objective measures compared with self-report [38] or maintained in the longer term [44]. Only one quasi-experimental study used a validated self-report measure [37], and this reported no significant differences in PA outcomes Table 5.

## Discussion

This review identified 23 interventions that used the HAPA model with the aim of increasing PA for people with long-term conditions (CHD, stroke, T2DM, mobility limitations, spinal cord injury, cancer, and obesity). The interventions utilized between three and nine HAPA variables, most commonly action planning (*n* = 22), coping planning (*n* = 20), intention (*n* = 20), and least commonly recovery self-efficacy (*n* = 5). The finding of high variability in numbers of HAPA model constructs used in interventions was noted in a published meta-analysis [15].

PA outcomes are improved when HAPA interventions are implemented, as noted in a 2019 systematic review [15]. However, in this review, less than half of the RCTs had significant intervention effects on PA. Further, the selection of type of PA measure varied across studies. Self-report and non-validated measures are a particular concern given the limited significance found. In addition, in the studies reviewed, it is not clear which HAPA-based intervention components led to the changes in PA and additional analysis is needed to make these determinations. Whether or not PA outcomes improved or not may have been related to factors not associated with the HAPA model itself; for example, sample characteristics, fidelity to the protocol by interventionists, circumstances related to the participant or environment, and how the HAPA constructs were operationalized.

It is suggested that self-efficacy is more important than other variables (intention, planning, and the health behavior) in predicting behavior change through the HAPA model [15]. Other reviews found that interventions that targeted self-efficacy and volitional variables were more likely to have positive effects [55]. Our review found that task self-efficacy was operationalized in 18/23 (78%) interventions, maintenance self-efficacy in 11/22 (50%), and recovery self-efficacy in only 5/22 (23%). Four interventions did not include any form of self-efficacy in the description of the intervention. Interestingly, for the two papers reporting nonsignificant PA outcomes [20, 27], one operationalized none of the three self-efficacy variables in the intervention [20] and the other only operationalized one (task self-efficacy) of the three self-efficacy variables [27]. The effects of including a few, or some, but not all HAPA model variables in the design of interventions are not fully understood. Questions remain about how many HAPA constructs must be operationalized in the intervention to be considered a HAPA-framed study [56]. Our review highlights that the selection of HAPA variables operationalized and the rationale for excluding variables must be addressed in future research reports to improve the interpretation of outcomes and transparency. The decision to use some but not all of the HAPA constructs raises the question, “how many HAPA constructs must be included to be considered adherent to the HAPA model?”.

Only three interventions [24, 30, 33] reported using the stage approach suggested within the HAPA model, meaning that in most studies, participants may have been offered strategies that may not be most effective to change behavior. Because few used the stage model format of HAPA, the current review did not find consensus about the value of the stage versus continuum model for PA interventions in long-term conditions. HAPA interventions designed using the stage model format will differentiate goal-setting activities (intention focus) and goal pursuit activities (behavior focus) [7]. Using HAPA as a stage model requires pre-assessment of participants to determine whether each is a pre-intender, intender, or actor. Once this is determined, the intervention components can be individually operationalized based on the stage of the participant to engage with the intervention. For example, building task self-efficacy is most relevant for pre-intenders and intenders, and enhancing maintenance and recovery self-efficacy is needed for actors who may experience barriers and interruptions to sustained PA [7]. Since most studies did not measure baseline assessments for stage, we were not able to discern the benefits of one approach over the other. For those studies that used a continuum approach, it is unclear why some HAPA variables were included and others not. Indeed, several papers that described interventions targeting PA did not include a PA outcome measure. Therefore, researchers need to provide a robust rationale for decisions made about exclusion of HAPA variables to allow their outcomes to be interpreted and translated. This review supports that increased consistency in intervention reporting is needed to better understand research on HAPA interventions as stated in other published reviews of HAPA interventions [56, 56].

Half (*n* = 12) of 23 interventions in our review used more than one modality to deliver content. Given the experiences during the recent COVID-19 pandemic, we expect a continued trend towards using more app and web-based modes of delivery for interventions aimed at health promotion [47]. During the pandemic, researchers were successful in transitioning in-person interventions to virtual formats though they reported that optimization of interventions depended on a robust system of stakeholder engagement, use of online software for data collection, and clear usage data tracking procedures [47]. Multiple modality strategies have been described as the concept “blended care” which combines therapeutic guidance in the form of telephone or in-person delivery with a digital component such as a website or app is growing in numbers [48]. Having multiple strategies for delivery may make it more challenging to determine which behavior change strategies lead to the desired outcome.

### Recommendations for Future Research

This review highlighted many opportunities for future HAPA model research. A wide variety of long-term conditions were included in our review. As HAPA research grows, reviews of PA interventions and how HAPA variables are operationalized for specific conditions will be useful. This review did not focus on differentiating PA outcomes based on a single vs. multiple modality approach; however, further research examining a “blended care” approach to PA interventions is needed in samples with long-term conditions. The use of “booster” doses or planned repetitions of the intervention was incorporated in several interventions consistent with literature that suggests “boosters” can mitigate relapses in the behavior change process [49]. Further research is needed to determine whether booster doses of HAPA intervention components will lead to better PA outcomes in persons with long-term conditions.

In the interest of transparency and scientific translation, we encourage authors to clearly report how the HAPA model is operationalized and, specifically, to state the rationale for excluding variables. The lack of detail in many studies and protocols reduced the extent to which we could provide more in-depth analyses. More research is needed to compare HAPA interventions for PA in long-term conditions that include differing HAPA constructs. By doing so, researchers will better understand the impact of intervention designs that operationalize only a portion of the HAPA model constructs versus all of them.

The benefits of using theory to frame interventions extend beyond the evaluation of effectiveness to provide clearer explanations of the interactions between the intervention as delivered and the outcomes experienced by the participants [46]. The ability to replicate interventions from published RCTs is desirable to promote translational science. However, data suggests that few papers provide adequate detail for replication to occur [50, 51]. In our review, only 12 interventions in 30 total papers provided sufficient protocol detail for replication, and our ability to report and analyze intervention details for this review was limited by these omissions. This finding highlights the need to adhere to established protocol and outcome study reporting requirements to enhance information exchange and translation.[100]

### Study Limitations and Strengths

To our knowledge, this is the first scoping review to describe in detail PA interventions developed using the HAPA model for PA in long-term conditions, how HAPA constructs were operationalized to promote PA in persons with various long-term conditions, and to provide assessments of quality. This review builds on other reviews that have examined the efficacy of HAPA interventions [15, 55, 56] and adds additional focus on PA interventions for long-term conditions. The PICO we used to guide our search focused on one theory (HAPA) and one lifestyle intervention and outcome (PA) and different long-term conditions. We acknowledge that long-term conditions are diverse in experience and challenges related to PA. While this review examined PA as the outcome, other lifestyle and behavioral factors affect long-term conditions and are important to consider in designing interventions. Research has shown that success in initiating and maintaining PA may be a gateway behavior that can promote other lifestyle behaviors important for specific long-term conditions [58]. Thus, isolating PA as an outcome for interventions may not achieve a broader impact on overall lifestyle for people living with long-term conditions. We encourage researchers to consider the benefits of broader lifestyle approaches.

We included protocols and one intervention development paper in our review to capture interventions with planned trials. We updated our search from the original review protocol with end date March 19, 2024, to be as inclusive as possible. In some cases, RCT and quasi-experimental papers referred to earlier published protocols; however, it is worth noting that protocols can change between publication and implementation. While we took care to extract details from each paper, when multiple theories were used and variables were not explicitly named for the HAPA model, we did not include them in the list of variables operationalized for that intervention. Therefore, it is possible that the included papers simply did not provide sufficient detail to identify included HAPA variables.

Small sample sizes, varied study designs, differences in distribution of sex across study samples, and the range of long-term conditions are examples of the heterogeneity of the papers included in this review thereby making it more difficult to make comparisons between interventions. While our search strategy attempted to include a wide range of long-term health conditions for which HAPA interventions are used to increase PA, this inclusive approach also makes it more challenging to compare interventions that target different populations.

## Conclusion

This review demonstrated positive effects of the HAPA on PA interventions for behavior change. However, the wide variability in the selection of HAPA variables that were operationalized meant comparisons between studies were not possible. A major outcome of this review is our finding that the HAPA model for intervention development in PA is not often implemented in its full scope. Researchers appear to pick and choose constructs to operationalize without clear rationale. More in-depth analysis is needed to clarify which HAPA constructs, settings, and modes of delivery lead to the desired PA behavior change and whether operationalizing all or only a few of the constructs is effective in the context of long-term conditions. Further, researchers must select PA measures that are validated, reliable, and as objective as possible. Doing so will allow clearer interpretation of findings and a greater likelihood that interventions are replicated. Lastly, few studies reported fidelity metrics for adherence to the intervention protocol which is necessary to fully interpret the quality of the intervention delivery and the outcomes achieved. Given the significant morbidity and mortality associated with long-term conditions and sedentary lifestyle, research continues to be needed that addresses promotion of sustained PA and other lifestyle behaviors that affect long-term conditions. As more HAPA interventions for specific populations are developed and tested, focused meta-analyses on specific long-term conditions will be possible. We believe this review may be of interest to researchers, policy makers, and health professionals to inform intervention development, the evaluation of HAPA intervention research, and translation of HAPA interventions to practice.

## Supplementary Information

Below is the link to the electronic supplementary material.

Supplementary file1 (DOCX 17.9 KB)

Supplementary file2 (PDF 368 KB)

Supplementary file3 (DOCX 46.9 KB)

## Data Availability

The datasets used and analyzed for this review are available as additional files.

## Abbreviations

CINAHL: Cumulative Index to Nursing and Allied Health Literature
HAPA: Health Action Process Approach
MeSH: Medical Subjects Headings
PA: Physical activity
PICO: Population, intervention, control, and outcome
PRISMA-ScR: PRISMA Extension for Scoping Review
RCT: Randomized controlled trial
