# Supplementary material for: Physical Activity Interventions Framed by the Health Action Process Approach for Adults with Long-Term Conditions: A Scoping Review
Source: Int J Behav Med. 2024 Jul 15;31(6):987–1017. doi: 10.1007/s12529-024-10305-2 (PMC11588932; doi:10.1007/s12529-024-10305-2)
Supplement: Supplementary file 1 — Supplementary file1 (DOCX 17.9 KB) [file 12529_2024_10305_MOESM1_ESM.docx]

Additional file 1

Aim: scoping review to examine the ways that the Health Action Process Approach (HAPA) constructs have been operationalized in exercise interventions targeting persons with chronic disease.

Objective: How have the elements, strategies and techniques targeting constructs of the HAPA model been defined and described for physical activity interventions

| Design | Population | Intervention | Comparator | Outcome |
| --- | --- | --- | --- | --- |
| Primary data from quantitative and qualitative studies from 1992 to August 2021 | Adults aged 18 years and over  Chronic Disease  Long term conditions  Non-communicable disease*  Chronic conditions  Lung or pulmonary conditions  Cardiovascular disease  Coronary heart disease  Diabetes mellitus  Endocrine conditions  Cardiovascular risk factors  Hypertension  Overweight  Obesity  Cancer  Nervous system diseases  Muscular diseases  Stroke or cerebrovascular accident  Liver disease  Kidney disease  Colon disease  Exclude psychiatric conditions | Physical activity  Physical activity therapy  Exercise  Exercise Therapy  Physical fitness  Physical*- (fit*or fitness or train* or therap* or activit*)  Physical activity rehabilitation (rehabilitat*)  Exercise rehabilitation (rehabilitat*)  Fitness exertion  Strength  Aerobic | For RCT studies - usual care | Health Action Process  Approach  HAPA  HAPA approach*  HAPA model*  HAPA theory  “Health Action Process Approach theory”  “Health Action Process Approach model” |
