# Supplementary material for: Physical Activity Interventions Framed by the Health Action Process Approach for Adults with Long-Term Conditions: A Scoping Review
Source: Int J Behav Med. 2024 Jul 15;31(6):987–1017. doi: 10.1007/s12529-024-10305-2 (PMC11588932; doi:10.1007/s12529-024-10305-2)
Supplement: Supplementary file 3 — Supplementary file3 (DOCX 46.9 KB) [file 12529_2024_10305_MOESM3_ESM.docx]

**Additional File 3: HAPA Scoping Review Article Selection**

Exclude: Children (<18 years of age); Mental health condition as target of HAPA physical activity/exercise intervention

Intervention Target: Physical Activity and/or Exercise

*More than one published paper about the intervention included in the review.

|  | **Author/Source** | **Title** | **Population:** Adults aged 18 and older with chronic health conditions | **Intervention:** PA/Exercise  Exposure = all research studies/methods, literature reviews, articles related to study design and intervention details | **Comparator:** | **Outcome**  PA intervention framed by Health Action Process Approach Concepts | **Meets Inclusion/**  **Exclusion criteria Yes/No** |
| --- | --- | --- | --- | --- | --- | --- | --- |
| 1 | Aliabad, H. O., et al. (2014). Global journal of health science 6(6): 198‐208.  RCT | "Maintenance of physical activity and exercise capacity after rehabilitation in coronary heart disease: a randomized controlled trial." | Adults  CAD  After Rehab | Booklet Intervention – promoting self-directed maintenance of PA | Usual care | Yes | Yes – Agreed by SM  14/10/2022 |
| 2 | Anderson AS, Dunlop J, Gallant S, et al BMJ Open 2018;8:e019410. doi: 10.1136/bmjopen-2017-019410 | Feasibility study to assess the impact of a lifestyle intervention (‘LivingWELL’) in people having an assessment of their family history of colorectal or breast cancer | Adults  Cancer | Intervention protocol – included PA |  | HAPA and others | No, unable  to differentiate HAPA constructs  Agree by SM  14/10/2022 |
| 3 | Berli et al. (2016)  Social Science and Medicine 163; 89-97  <http://dx.doi.org/10.1016/j.socscimed.2016.07.003>  RCT for Scholz protocol | "A Dyadic Action Control Trial in Overweight and Obese Couples (DYACTIC)." | Adults  Obesity | Intervention | Control group | HAPA | Yes  Agreed by SM 06/03/2023 |
| 4 | Chiu, C. Y., et al. (2011). Rehabilitation psychology 56(3): 171-181.  Chiu et al (2021). <https://clinicaltrials.gov/ct2/show/NCT05124522> | "The Health Action Process Approach as a Motivational Model for Physical Activity Self-Management for People With Multiple Sclerosis: A Path Analysis." | Adults  MS (was this a population in the PICO?) | No PA intervention – dietary self-management validation study | N/A |  | No  HAPA clinical trial registered without intervention details  Agreed by SM 14/10/2022 |
| 5 | Crhova, M. et al. (2022). Klinicka Onkologie  35 (5), pp. 392-400  Abstract only; full-text not in English | “Exercise as a tool to improve the quality of life of patients after breast cancer treatment” | Adults  Cancer | PA intervention | Control | Yes | No  Agreed by SM 02/04/2023 |
| 6 | Daryabeygi-Khotbehsara, R., et al. (2021). International journal of clinical practice 75(5): e1399  RCT | "Short-term effectiveness of a theory-based intervention to promote diabetes management behaviours among adults with type 2 diabetes in Iran: A randomised control trial." | Adults  T2DM | Intervention | Control group with no intervention; offered nutrition counseling after completion of study | Yes | Yes  Agreed by SM 14/10/2022 |
| 7 | Dennett, A. M. et al. (2022). JMIR Research Protocols | “Efficacy of Group Exercise-based cancer rehabilitation Delivered via Telehealth: Protocol for a randomized controlled trial” | Adults  Cancer | Intervention | Usual Care | No; HAPA measure used but theory not used in intervention design | No  Agreed by SM 02/04/2023 |
| 8 | Ludwig, Kurderer, Dettmers, C. (2016). Neurologie und rehabilitation 22(1): 43-52 | "Application of volitional training strategies in neurological rehabilitation to increase regular walking training -a pilot study." | Only in German |  |  |  | No  Not in English  Agreed by SM  14/10/2022 |
| 9 | Döbler, A., et al. (2018). Rehabilitation psychology 63(2): 170-181.  RCT | "Telephone-delivered lifestyle support with action planning and motivational interviewing techniques to improve rehabilitation outcomes." | Adults  T2DM | Telephone intervention to support self-managed PA | Usual care | Yes | Yes  Agreed by SM  14/10/2022 |
| 10 | Dohnke, B., et al. (2010). Research in sports medicine (Print) 18(4): 219-235. | "Motivation and participation in a phase III cardiac rehabilitation programme: an application of the health action process approach." | Adults  Phase III cardiac rehab | No intervention - study looking at people who attended/adhered to CR | N/A | Yes | No  Agreed by SM  14/10/2022 |
| 11 | Duan, Y. P., et al. (2018). Journal of medical Internet research 20(11): e12052.  Pilot RCT | "Evaluation of a Web-Based Intervention for Multiple Health Behavior Changes in Patients With Coronary Heart Disease in Home-Based Rehabilitation: Pilot Randomized Controlled Trial." | Adults  CHD | Intervention – 8-week PA & Fruit & Veg module | Waiting list | Yes | Yes  Agree by SM 14/10/2022 |
| 12 | Feng, D. et al. (2022). Reviews in Cardiovascular Medicine, 23(2), 64. | Attitudes and behavioral intentions of aortic dissection survivors towards exercise: An application of the health action process approach” | Adults with Aortic Dissection | No intervention –  Qualitative research | N/A | N/A | No  Agree by SM 02/04/2023 |
| 13 | Foulon, B. L. and K. A. Ginis (2013). Disability and rehabilitation 35(24): 2073‐2080.  RCT | "The effects of physical activity vignettes on physical activity-related social cognitions among people with spinal cord injury." | Adults  SCI | Intervention – HAPA informed portrait vignettes | Non-tailored vignettes | Yes | Yes  Agree by SM  14/10/2022 |
| 14 | *Ghisi, G. L. d. M., et al. (2015). Patient education and counseling 98(5): 612-621.  Quasi-experiment | "Behavior determinants among cardiac rehabilitation patients receiving educational interventions: an application of the health action process approach." | Adults  Cardiac Rehab | Educational intervention including PA as target behavior | Non-HAPA education | Yes | Yes  Agreed by SM  14/10/2022 |
| 15 | *Ghisi, G. L. d. M., et al. (2015). Heart & Lung: the journal of critical care 44(6): 474-480.  Quasi-experiment | "Knowledge and exercise behavior maintenance in cardiac rehabilitation patients receiving educational interventions." | Adults CR | Educational intervention – including PA | Non HAPA education | Yes | Yes  Agreed by SM  14/10/2022 |
| 16 | Ghisi, G. L. d. M., et al. (2021). BMC public health 21(1): 1236. | "Patient education program for Brazilians living with diabetes and prediabetes: findings from a development study." | Adults  T2DM | No intervention |  | Yes and other theories | No  Agreed by SM  22/11/2022 |
| 17 | Greaves, C., et al. (2015). The international journal of behavioral nutrition and physical activity 12: 1.  Pilot RCT | "Waste the waist: a pilot randomised controlled trial of a primary care based intervention to support lifestyle change in people with high cardiovascular risk." | Adults  High CV Risk | Intervention – group based weight management intervention including diet and PA | Usual care | Yes | Yes  Agreed by SM  22/11/2022 |
| 18 | Hardcastle, S. J., et al. (2019). BMJ open 9(5): e028369.  Protocol only | "Promoting physical activity in regional and remote cancer survivors (PPARCS) using wearables and health coaching: randomised controlled trial protocol." | Adults  Cancer | Intervention protocol – fitbit charge activity tracker and six sessions of heath coaching | Control group | Yes | Yes  Agreed by SM  22/11/2022 |
| 19 | *Hardcastle, S. J., et al. (2021). Journal of cancer survivorship: research and practice. | "Predicting physical activity change in cancer survivors: an application of the Health Action Process Approach." | Adults  Cancer | Model testing only this paper; intervention is WAATAP (Maxwell-Smith RCT) |  | Yes | No, model testing |
| 20 | Hardcastle, S.J., et al (2022) Journal of Cancer Survivorship, 16(6, 1176-1183 | “Predicting physical activity change in cancer survivors: an application of the Health Action Process Approach” | Adults  Cancer | Model testing only; see #19 | N/A | N/A | No, model testing.  Agreed by SM 02/04/2023 |
| 21 | Hattar, A., et al. (2016). Applied psychology. Health and well-being 8(1): 127-151. | "Predicting Physical Activity-Related Outcomes in Overweight and Obese Adults: A Health Action Process Approach." | Adults  Obesity | No Intervention – qualitative research | N/A |  | No  Agreed by SM  22/11/2022 |
| 22 | Hinrichs, T., et al. (2011). Trials 12: 263.  Protocol only | "Effects of an exercise programme for chronically ill and mobility-restricted elderly with structured support by the general practitioner's practice (HOMEfit) - study protocol of a randomised controlled trial." | Adults  Chronic disease | Intervention protocol – home-based exercise programme | Control group | Yes | Yes  Agreed by SM  22/11/2022 |
| 23 | Hinrich et al. (2016)  Journal of the American Geriatrics Society. 2016 Nov;64(11):2270-9. DOI: 10.1111/jgs.14392  RCT | “Home‐Based Exercise Supported by General Practitioner Practices: Ineffective in a Sample of Chronically Ill, Mobility‐Limited Older Adults (the HOME fit Randomized Controlled Trial)” | Adults  Chronic disease | Intervention protocol – home-based exercise programme | Control group | Yes | Yes  Agreed by SM  22/11/2022 |
| 24 | Ho, J. W. C., et al. Journal of Cancer Survivorship (2020) 14:424–433  RCT acceptability/feasibility | "Moving Bright, Eating Smart"- A phase 2 clinical trial on the acceptability and feasibility of a diet and physical activity intervention to prevent recurrence in colorectal cancer survivors." | Adults  Colorectal cancer | Intervention protocol – PA intervention group and a dietary intervention group | Usual Care | HAPA and TPB | Yes  Agreed by SM  22/11/2022 |
| 25 | Karthijekan, K. & Cheng, H.Y. (2022). PloS One, 17(7)  Protocol only | Effectiveness of a motivated, action-based intervention on improving physical activity, exercise self-efficacy, and cardiovascular risk factors of patients with coronary heart disease in Sri Lanka: A randomized controlled trial protocol. | Adults  CHD | Culture-specific, motivated,  and action-based intervention to increase PA | Usual Care | HAPA | Yes  Agreed by SM 02/04/2023 |
| 26 | Knoll, N., et al. (2018). BMC musculoskeletal disorders 19(1): 221.  RCT | "Facilitating physical activity and reducing symptoms in patients with knee osteoarthritis: study protocol of a randomized controlled trial to test a theory-based PrevOP-psychological adherence program (PrevOP-PAP)." | Adults  Knee osteoarthritis | Intervention – includes PA | Active control | HAPA | Yes  Agreed by SM 22/11/2022 |
| 27 | Lightfoot, C.J. et al. (2022). Journal of Medical Internet Research | “The Codevelopment of “My Kidneys & Me”: A digital self-management program for people with chronic kidney disease” | Adults  CKD | No intervention/de-velopment of a resource for patients | N/A | Multiple theories | No  Agreed by SM 02/04/2023 |
| 28 | Lim, S., et al. (2017). Diabetes Research & Clinical Practice 126: 254-262. | "Comparing a telephone- and a group-delivered diabetes prevention program: Characteristics of engaged and non-engaged postpartum mothers with a history of gestational diabetes." | Adults  Gestational DM | Intervention – lifestyle modification program. Group –v- telephone |  | HAPA | No  Agreed by SM  22/11/2022 |
| 29 | Lippke, S. and R. C. Plotnikoff (2014). Health psychology : official journal of the Division of Health Psychology, American Psychological Association 33(1): 77-84. | "Testing two principles of the Health Action Process Approach in individuals with type 2 diabetes." | Adults  T2DM | No intervention – questionnaire |  | HPA | No  Agreed by SM  22/11/2022 |
| 30 | Liu et al., 2019. Phys Ther. 99:1616–1627.  Protocol only | "Individualized Exercise Program Plus Behavioral Change Enhancement Strategies for Managing Fatigue in Frail Older People." | Adults  Frail older adults | Intervention | No behavior change component | HAPA | Yes  SM 22/11/2022 |
| 31 | *Ma, J et al (2020) Spinal Cord, 58(7), 778-786  Intervention development description | Co-development of a physiotherapist-delivered physical activity intervention for adults with spinal cord injury | Adults  SCI | Intervention – co-design process of a PA intervention | N/A | HAPA | Yes  Agreed by SM  22/11/2022 |
| 32 | *Ma, West, & Martin Ginis (2019).  Sports Medicine (2019) 49:1117–1131  <https://doi.org/10.1007/s40279-019-01118-5>  RCT | The Effects of a Patient and Provider Co‑Developed, Behavioral  Physical Activity Intervention on Physical Activity, Psychosocial  Predictors, and Fitness in Individuals with Spinal Cord Injury:  A Randomized Controlled Trial | Adult SCI | Intervention – PT session followed by 8 x 15 minute PA coaching session | Control matched by activity level | HAPA | Yes  Agreed by SM 06/03/2023 |
| 33 | *Maxwell-Smith, C., et al. (2018). International journal of clinical and health psychology : IJCHP 18(2): 124-132.  Protocol for #34 RCT | "Wearable Activity Technology And Action-Planning (WATAAP) to promote physical activity in cancer survivors: Randomised controlled trial protocol." | Adults  Cancer | Intervention – wearable trackers | Information only control group | HAPA | Yes  Agreed by SM 06/03/2023 |
| 34 | Maxwell-Smith C, Hince D, Cohen PA, Bulsara M, Boyle T, Platell C, Tan P, Levitt M, Salama P, Tan J, Salfinger S. Psycho-Oncology. 2019 Jul;28(7):1420-9. doi/10.1002/pon.5090  RCT | A randomized controlled trial of Wearable Activity Technology And Action-Planning (WATAAP) to Promote Physical Activity in Colorectal and Endometrial Cancer Survivors. | Adults Cancer | Intervention-  Wearable trackers | Information only control group | HAPA | Yes  Agreed by SM  22/11/2022 |
| 35 | McCleary, N., et al. (2020). Health psychology : official journal of the Division of Health Psychology, American Psychological Association 39(12): 1048-1061. | "Interventions supporting cardiac rehabilitation completion: Process evaluation investigating theory-based mechanisms of action." | Adults  Cardiac Rehabilitation | Cardiac rehab processes to increase attendance |  | HAPA | No  Agreed by SM 22/11/2022 |
| 36 | Meadows, R. and R. J. Paxton (2018). Journal of immigrant and minority health 20(1): 147-154. | "Stage Validity of the Health Action Process Approach in African American Breast Cancer Survivors." | Adult  Cancer | No intervention; stage validity |  |  | No  Agreed by SM  22/11/2022 |
| 37 | Mohammadi Zeidi, I., et al. (2020). Nursing open 8(1): 442-452. | "Predicting psychological factors affecting regular physical activity in hypertensive patients: Application of health action process approach model." | Adult  Hypertension | No intervention |  |  | No  Agreed by SM  22/11/2022 |
| 38 | Murphy, M. L., et al. (2019). Contemporary clinical trials 78: 53-62. | "Preliminary efficacy and feasibility of referral to exercise specialists, psychologists and provision of a technology-based behavior change support package to promote physical activity in school teachers 'at risk' of, or diagnosed with, type 2 diabetes: The 'SMART Health' Pilot Study Protocol." | Adult  T2DM | Intervention - PA | Wait list control | HAPA and others; blended theories | No  Agreed by SM 06/03/2023 |
| 39 | Namadian, M., et al. (2016). Psychology of Sport & Exercise 26: 83-93. | "Motivational, volitional and multiple goal predictors of walking in people with type 2 diabetes." | Adults  T2SM | No intervention |  |  | No  Agreed by SM  22/11/2022 |
| 40 | O'Brien, J., et al. (2018). Trials 19.  Protocol only | "Improving physical activity, pain and function in patients waiting for hip and knee arthroplasty by combining targeted exercise training with behaviour change counselling: study protocol for a randomised controlled trial." | Adults  Osteoarthritis | Intervention – individualized programme of exercise and counselling | Usual care | HAPA | Yes  Agreed by SM  22/11/2022 |
| 58 | Parkinson, J. et al. (2023) Health Promotion International 38: 1-12. | “Health action process approach: promoting physical activity, and fruit and vegetable intake among Australian adults” | Adults; no chronic condition specifed | 4 program sessions | No control | HAPA | No  Agreed by SM 19/04/2024 |
| 41 | Parschau, L., et al. (2014). Rehabilitation psychology 59(1): 42-49. | "Physical activity among adults with obesity: testing the Health Action Process Approach." | Adults  Obesity | No Intervention |  |  | No  Agreed by SM  22/11/2022 |
| 42 | Paxton, R. J. (2016). Psycho-oncology 25(6): 648-655. | "The health action process approach applied to African American breast cancer survivors." | Adults  Cancer | No intervention |  |  | No  Agreed by SM  22/11/2022 |
| 43 | Plaete, J., et al. (2015). JMIR research protocols 4(4): e141.  Degroote et al (2018) | "A Self-Regulation eHealth Intervention to Increase Healthy Behavior Through General Practice: Protocol and Systematic Development." | Adults | Intervention – self regulation e-health |  | HAPA | No  No chronic condition  No Agreed by SM  22/11/2022 |
| 44 | Platter, M., et al. (2016). Wiener klinische Wochenschrift 128(5-6): 175-181.  Quasi-experiment | "Supporting cardiac patient physical activity: a brief health psychological intervention." | Adults  CAD | Intervention– 3 part intervention incl. personal action plan & coping pan | Usual care | HAPA | Yes  Agreed by SM  22/11/2022 |
| 45 | Plotnikoff, R. C., et al. (2017). *Preventive medicine*, *105*, 404-411.  RCT = Wilczynska protocol | Integrating smartphone technology, social support and the outdoor physical environment to improve fitness among adults at risk of, or diagnosed with, type 2 diabetes: findings from the ‘eCoFit’random-ized controlled trial. | At risk or diabetes Mellitus | Intervention | Waitlist control | HAPA and others | Yes  Agreed by SM 06/03/2023 |
| 46 | Poppe, L., et al. (2019). Journal of medical Internet research 21(8): e13363.  RCT | "Efficacy of a Self-Regulation-Based Electronic and Mobile Health Intervention Targeting an Active Lifestyle in Adults Having Type 2 Diabetes and in Adults Aged 50 Years or Older: Two Randomized Controlled Trials." | Adults  T2DM | Intervention | Waitlist control | HAPA | Yes  Agreed by SM  22/11/2022 |
| 47 | *Reinwand, D., Kuhlmann, T., Wienert, J., de Vries, H., & Lippke, S. (2013). *BMC Public Health*, *13*, 1081. <https://doi-org.libproxy.uccs.edu/10.1186/1471-2458-13-1081>  Protocol for Storm et al. RCT (2016) | Designing a theory- and evidence-based tailored eHealth rehabilitation aftercare program in Germany and the Netherlands: study protocol. | Adults  CAD-after cardiac rehab | Intervention - | Waiting list control | HAPA | Yes  Agreed by SM  22/11/2022 |
| 48 | Scholz, U. and C. Berli (2014). BMC public health 14: 1321.  Protocol for Berli RCT | "A Dyadic Action Control Trial in Overweight and Obese Couples (DYACTIC)." | Adults  Obesity | Intervention | Control group | HAPA | Yes  Agreed by SM  22/11/2022 |
| 49 | Schwarzer, R., et al. (2011). Rehabilitation psychology 56(3): 161-170. | "Mechanisms of Health Behavior Change in Persons With Chronic Illness or Disability: The Health Action Process Approach (HAPA)." | Adults | No intervention |  | HAPA | No  Agreed by SM  22/11/2022 |
| 50 | Sequeira et al., (2022)  <http://dx.doi.org.libproxy.uccs.edu/10.1007/s12529-022-10140-3> | Predicting Physical Activity in survivors of breast cancer: The health action process approach at the intra personal level | Adults  Breast Cancer | No intervention |  |  | No |
| 59 | Smith, S et al. (2023) BMJ Open Access  13:e068003 | “Promoting adherence to stroke secondary prevention behaviours by imparting behavior change skills: protocol for a single-arm pilot trial of Living Well After Stroke | Stroke survivors | Protocol for intervention | No control group | HAPA | Yes  Agreed by SM 19/04/2024 |
| 51 | Steca, P., et al. (2017). Psychology & health 32(3): 361-380. | "Changes in physical activity among coronary and hypertensive patients: A longitudinal study using the Health Action Process Approach." | Adults  HTN, CAD | No intervention |  |  | No  Agreed by SM  22/11/2022 |
| 52 | *Storm, V., et al. (2016). Journal of medical Internet research 18(4): e78.  RCT for Reinwand protocol | "Effectiveness of a Web-Based Computer-Tailored Multiple-Lifestyle Intervention for People Interested in Reducing their Cardiovascular Risk: A Randomized Controlled Trial." | Adults  CV Risk | Intervention | Waiting list control | HAPA | Yes  Agreed by SM  22/11/2022 |
| 53 | Ströbl, V., et al. (2013). Journal of rehabilitation medicine 45(2): 198-205.  RCT | "A combined planning and telephone aftercare intervention for obese patients: effects on physical activity and body weight after one year." | Adults  Obesity | Intervention | Standard care | HAPA | Yes  Agreed by SM  22/11/2022 |
| 54 | Wienert, J., et al. (2017). International journal of behavioral medicine 24(6): 908-914. | "Motivational and Volitional Correlates of Physical Activity in Participants Reporting No, Past, and Current Hypertension: Findings from a Cross-Sectional Observation Study." | Adults  HTN | No intervention |  |  | No  Agreed by SM  22/11/2022 |
| 55 | Wilczynska, M., et al. (2016). Contemporary clinical trials 49: 116-125.  Protocol for Plotnikoff RCT | “Rationale and study protocol for the ‘eCoFit’ randomized controlled trial:  Integrating smartphone technology, social support and the outdoor  physical environment to improve health-related fitness among adults at  risk of, or diagnosed with, Type 2 Diabetes” | Adults  T2DM | Intervention | Wait list control | HAPA + others | Yes  Agreed by SM  22/11/2022 |
| 56 | Zeidi, I. M., et al. (2021). Nursing open 8(1): 442-452. | "Predicting psychological factors affecting regular physical activity in hypertensive patients: Application of health action process approach model." | Adults  HTN | No intervention |  |  | No  Agreed by SM  22/11/2022 |
| 57 | Zhang et al., (2022). Frontiers in Psychology | “Theoretical explanation of upper limb functional exercise and its maintenance in postoperative patients with breast cancer” | Adults  Breast Cancer | No intervention | N/A |  | No  Agreed by SM 02/04/2023 |
